# Experimental evolution of defense against a competitive mold confers reduced sensitivity to fungal toxins but no increased resistance in *Drosophila *larvae

**DOI:** 10.1186/1471-2148-11-206

**Published:** 2011-07-14

**Authors:** Monika Trienens, Marko Rohlfs

**Affiliations:** 1 Zoological Institute, Department of Evolutionary Ecology and Genetics, Christian-Albrechts-University of Kiel, Am BotanischenGarten 1-9, D-24098 Kiel, Germany; 2 J.F Blumenbach Institute of Zoology and Anthropology, Animal Ecology, Georg-August-University of Göttingen, BerlinerStraße 28, D-37073 Göttingen, Germany

## Abstract

**Background:**

Fungal secondary metabolites have been suggested to function as chemical defenses against insect antagonists, i.e. predators and competitors. Because insects and fungi often compete for dead organic material, insects may achieve protection against fungi by reducing sensitivity to fungal chemicals. This, in turn, may lead to increased resistance allowing insects better to suppress the spread of antagonistic but non-pathogenic microbes in their habitat. However, it remains controversial whether fungal toxins serve as a chemical shield that selects for insects that are less sensitive to toxins, and hence favors the evolution of insect resistance against microbial competitors.

**Results:**

To examine the relationship between the ability to survive competition with toxic fungi, sensitivity to fungal toxins and resistance, we created fungal-selected (FS) replicated insect lines by exposing *Drosophila melanogaster *larvae to the fungal competitor *Aspergillus nidulans *over 26 insect generations. Compared to unselected control lines (UC), larvae from the FS lines had higher survival rates in the presence of *A. nidulans *indicating selection for increased protection against the fungal antagonist. In line with our expectation, FS lines were less susceptible to the *A. nidulans *mycotoxin Sterigmatocystin. Of particular interest is that evolved protection against *A. nidulans *and Sterigmatocytin was not correlated with increased insect survival in the presence of other fungi and mycotoxins. We found no evidence that FS lines were better at suppressing the expansion of fungal colonies but observed a trend towards a less detrimental effect of FS larvae on fungal growth.

**Conclusion:**

Antagonistic but non-pathogenic fungi favor insect variants better protected against the fungal chemical arsenal. This highlights the often proposed but experimentally underexplored importance of secondary metabolites in driving animal-fungus interactions. Instead of enhanced resistance, insect larvae tend to have evolved increased tolerance of the fungal competitor. Future studies should examine whether sensitivity to allelopathic microbial metabolites drives a trade-off between resistance and tolerance in insect external defense.

## Background

The fungal kingdom contains an underexplored diversity of organisms occurring in almost all terrestrial, marine and freshwater ecosystems [1]. Because fungi constitute an important food source for animals or act as serious competitors of them [2,3] they are assumed to have evolved defense strategies employing toxic or deterrent secondary chemicals [4-6]. Animals, in turn, may be selected for counter-adaptations that render fungal toxins less detrimental [7], thus placing a higher selective pressure on fungi, and possibly fueling a co-evolutionary process.

In terrestrial decomposer communities the larval stages of many saprophagous insect species seem to be engaged in competition with filamentous fungi for dead organic material. For instance, while some microbes, such as bacteria and yeast fungi, are essential food sources for insect larvae foraging on dead organic material [8,9] there are negative relationships between the occurrence of filamentous fungi in the larval habitat and insect development, e.g. in sciarid fly larvae [10], *Necrophorus*beetle larvae [11], house fly larvae [12,13] and *Drosophila *fly larvae [14,15]. This type of insect-fungus interaction is, however, not unidirectional but involves complex reciprocal consequences as insect larvae can seriously hamper fungal development. In saprophagous drosophilid flies breeding on rotting plant tissue, particularly, reciprocal impairment in larval-fungus interactions is wide-spread [16-21]. These reciprocal fitness consequences strongly suggest a process of interspecific competition between insect and filamentous fungi. In line with the definition of interspecific competition high larval density negatively influences fungal growth and benefits the *Drosophila *larvae [19]. Moreover, if the fungi establish before the larvae they appear to become competitively superior over the insects (priority effect). When this occurs colony growth and reproduction are less negatively affected by the insects and *Drosophila *larvae suffer significantly higher mortality rates than when fungi and flies establish at the same time [18,19].

The mechanism underlying the reduction of insect fitness in the presence of competing fungi may involve the secretion of toxic fungal secondary metabolites (mycotoxins) into the larval feeding substrate, a fungal trait that has been shown to be under tight genetic regulation [22]. This mechanism could function because mycotoxins are effective against *Drosophila *larvae in pharmaceutical tests [23]. Moreover, deleting a gene encoding a regulatory protein involved in positively influencing secondary metabolite expression in various filamentous fungi competing with *Drosophila *larvae was beneficial to the insects and detrimental to the fungi [15]. Thus, filamentous fungi may be considered as allelopathic organisms that build a chemical shield against saprophagous insect larvae. *Drosophila *populations harbor heritable variation in their ability to withstand the proposed fungal chemical shield because, more than 20 years ago, Melone and Chinnici [24] were able to select larval *D. melanogaster *populations for reduced sensitivity to the mycotoxins, such as Aflatoxin B1, that filamentous fungi secrete into the surrounding environment. Recently, we found heritable variation in the ability of *Drosophila *larvae to develop successfully in the presence of a competing filamentous fungus [25]. From the results of these studies we derived the key hypothesis of this paper: heritable variation in the competitive ability of saprophagous *D. melanogaster *populations against filamentous fungi is due to variation in their ability to cope with toxic fungal metabolites. We would therefore expect a positive correlation between larval survival in the presence of the competing mold and survival in substrates contaminated with fungus mycotoxins. If insect adaptation to fungal competitors involves a counter-adaptation to the fungal chemical shield proposed, we would also expect that larvae with such adaptations would impair mold development more than unadapted larvae. Such an increase in insect competitive ability or resistance may place a greater selective pressure on fungi and thus has the potential to fuel co-evolutionary dynamics in antagonistic animal-fungus interactions, i.e. arms races or Red Queen dynamics [26,27]. Alternatively, insect larvae may be selected for increased tolerance, that is, the ability to limit the negative effects of competition. Compared to resistance (i.e. competitor control), evolution of increased tolerance (i.e. damage control) is assumed not to correlate with enhanced negative effects on the fungus [28]. Thus, evolution of insect tolerance mechanisms is not expected to directly favor fungal counter-measures, e.g. more toxic secondary chemicals.

We use the *Drosophila*-*Aspergillus *system [15] as an ecological model to explore the relationship between variation in protection against fungal competitors and sensitivity to secondary metabolites by means of an experimental evolution approach. We therefore generated fungal selected (FS) and unselected control (UC) insect lines. The FS lines were created by forcing *Drosophila *to compete with toxin-producing fungus *Aspergillus nidulans *and so subjecting them to selective pressure. After 26 generations we stopped the selection and investigated first whether selection pressure imposed by the fungus had resulted in the evolution of enhanced *Drosophila *developmental success under moldy conditions. We secondly tested whether protection evolved against *A. nidulans *was due to mechanisms that give cross-protection against other filamentous fungi (*A. fumigatus *and *A. flavus*) or whether it only confers the ability to compete against *A. nidulans*. In a third experiment, we tested whether FS larvae were less susceptible than UC larvae to Sterigmatocystin, the most prominent mycotoxin secreted by *A. nidulans*. Moreover, we tested for cross-protection against other polyketide mycotoxins, Aflatoxin B1 and Ochratoxin A. In our fourth experiment we studied the effect of FS and UC lines on fungal growth to test for correlated responses in the ability of larvae to control the spread of the fungus.

## Results

### Protection against *A. nidulans *and cross-protection to other Aspergilli

**Figure 1:**
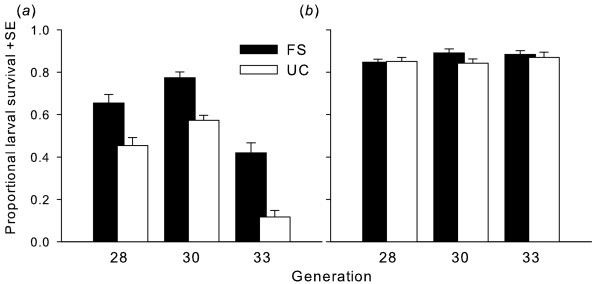
**Survival of *Drosophila *larvae selected by experimental evolution with competing *Aspergillus nidulans *or unselected**. Effect of selection with *A. nidulans *on the survival of *D. melanogaster *larvae to the adult stage after selection pressure was stopped at generation 26. Solid and open bars depict mean proportional larval survival of fungal selected (FS) and unselected control (UC) lines, respectively, under (*a*) moldy and (*b*) mold-free conditions (** *p *< 0.01; n.s. not significant; statistical details in Table 1).

We found a significant effect of the selection regime on the survival of larval *Drosophila *when the feeding substrate was infested with proliferating colonies of *A. nidulans*. FS *Drosophila *larvae had a significantly higher probability of surviving to the adult stage than UC larvae (Figure 1, Table 1). Although there was large variation between generations, the differences between survival rates of larvae from the FS and the UC lines remained fairly constant (20-30%) (Figure 1, Table 1). Moreover, the effects of selection history persisted after the last selection cycle at generation 26. There were no differences in larval survival between FS and UC populations under mold-free conditions (Figure 1, Table 1).

**Table 1:** *Drosophila *larval survival in the presence of *Aspergillus nidulans*

	with *A. nidulans*		mold-free control	
	
effect	*F*	*p*	*F*	*p*
selection regime (FS vs. UC)	*F*_1,4 _= 42.15	0.0029	*F*_1,4 _= 0.48	0.5268
generation	*F*_2,8 _= 70.48	< 0.0001	*F*_2,8 _= 0.45	0.6532
selection regime × generation	*F*_2,8 _= 4.19	0.0569	*F*_2,8 _= 0.71	0.5201

Generalized linear mixed model analysis of the factors affecting larval survival of *Drosophila melanogaster *dependent on the selection regime and insect generation in the presence of *Aspergillus nidulans *and under mold-free conditions.

FS: fungal selected *Drosophila *lines, UC: unselected control lines.

Larvae from the FS lines reared in the presence of the model fungal competitor, *A. fumigatus *had no higher survival than larvae from UC lines (FS mean = 0.23, SE = 0.03; UC mean = 0.16, SE = 0.03, *F*_1,4_ = 2.25, *p *= 0.2080). This was also true for FS larvae with *A. flavus *(FS mean = 0.24, SE = 0.03; UC mean = 0.17, SE = 0.03, *F*_1,4_ = 0.80, *p *= 0.4206).

### Sensitivity to Sterigmatocystin and other mycotoxins

**Figure 2:**
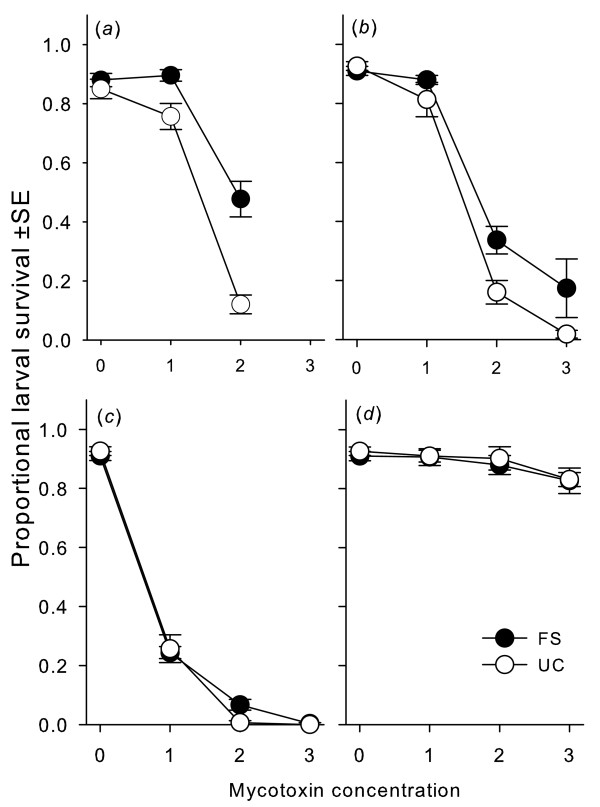
**Effect of mycotoxins on selected and unselected *Drosophila *larvae**. Effect of selection with *A. nidulans*on the survival of *D. melanogaster *larvae to the adult stage on substrate contaminated with Sterigmatocystin at generation 30 (*a*) and 33 (*b*), Aflatoxin B1 (*c*)and Ochratoxin A (*d*) (both at generation 33) at varying concentrations (μg/ml substrate). Dark symbols depict mean proportional larval survival of fungal selected (FS) and open symbols unselected control (UC) lines (see text and Table 2 for statistical details).

The FS populations were less susceptible to Sterigmatocystin than the UC populations (Figure 2a). Moreover, as indicated by a significant statistical interaction between 'selection regime' and'Sterigmatocystin concentration', larval survival dropped more rapidly in UC lines than in FS lines (selection regime: *F*1,4 = 11.65, *p *= 0.0270; concentration:*F*_2,8_ = 145.21, *p *< 0.0001; selection regime × concentration: *F*_2,8_ = 12.87, *p *= 0.0032). We repeated the toxin confrontation experiment with Sterigmatocystin in generation 33 and additionally tested sensitivity to Aflatoxin B1 and Ochratoxin A. These are two cytotoxic and carcinogenic mycotoxins that are not synthesized by *A. nidulans*, but Sterigmatocystin is the penultimate precursor of Aflatoxin B1 in *A. flavus *and thus of similar chemical structure [29]. *Drosophila *larvae showed mycotoxin specific survival patterns that depended significantly on the selection regime (Table 2; Figure 2b-c). However, post hoc analyses of larval survival for each mycotoxin revealed a significant effect of selection only for Sterigmatocystin (selection regime: *F*_1,4_ = 7.16,*p *= 0.0555; concentration: *F*_3,12_ = 126.12, *p *< 0.0001; selection regime × concentration: *F*_3,12_ = 5.29,*p *= 0.0148. There was not such an effect for Aflatoxin B1 (selection regime: *F*_1,4_ = 0.29, *p *= 0.6174;concentration: *F*_3,12_ = 216.03, *p *< 0.0001; selection regime × concentration: *F*_3,12_ = 1.63, *p *= 0.2337) or Ochratoxin A (selection regime: *F*_1,4_ = 0.49, *p *= 0.5233; concentration: *F*_3,12_ = 7.33, *p *= 0.0047; selection regime × concentration: *F*_3,12_ = 0.11, *p *= 0.9555).

**Table 2:** *Drosophila *larval survival as affected by mycotoxins

Effect	*F*	*p*
selection regime (FS vs. UC)	*F*_1,4 _= 3.54	0.1330
mycotoxin identity (myc.)	*F*_2,8 _= 162.12	< 0.0001
mycotoxin concentration (conc.)	*F*_3,12 _= 184.14	< 0.0001
selection regime × myc.	*F*_2,8 _= 6.44	0.0216
selection regime × conc.	*F*_3,12 _= 3.70	0.0429
myc. × conc.	*F*_6,24 _= 75.19	< 0.0001
selection regime × myc. × conc.	*F*_6,24 _= 3.01	0.0245

Generalized linear mixed model analysis of the factors affecting larval survival of *Drosophila melanogaster *dependent on the selection regime and mycotoxin identity (myc.) and mycotoxin concentration (conc.).

FS: fungal selected *Drosophila *populations, UC: unselected control populations.

### Effects on fungal performance

**Figure 3:**
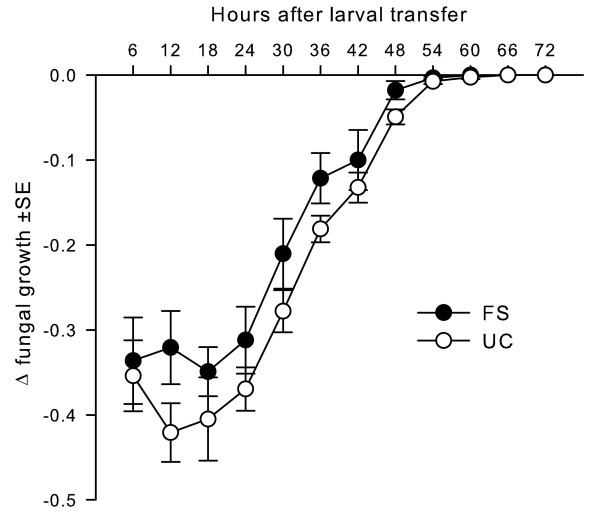
**Suppression of fungal growth by selected and unselected *Drosophila *larvae**. Effect of *Drosophila *larvae from the fungal selected (FS) and unselected (UC) lines on growth of *A. nidulans*. Solid and open symbols depict the mean proportional impairment of challenged colonies compared to unchallenged ones when confronted with larvae from FS or UC lines (see text and Table 3 for statistical details).

*Drosophila *larvae cause serious damage to *Aspergillus *colonies in this insect-fungus competition. The magnitude of this damage indicates the insect's competitive ability. We hypothesized that fly populations harboring a higher proportion of better protected variants (FS populations) should hamper fungal colonies to a higher degree than more vulnerable populations (UC populations). In contrast to our expectation of evolved resistance (i.e. *Drosophila *larvae developing more successfully in the presence of *A. nidulans *through intensified fungal colony suppression) there was no such effect on the fungal competitor (Figure 3, Table 3). Fungal colony development, the increase in substrate area occupied, is strongly hampered during the early stage of this interference competition (Δ fungal growth values below 0), followed by a period of recovery (Δ fungal growth values approach 0). The recovery may be due to alterations in larval foraging behavior when larvae switch from "surface feeding" to "digging", which may release fungal colonies from insect attack at the substrate surface. During the early phase it appears as if UC larvae had a more negative effect on fungal growth than those from the FS populations (Figure 3), however, the overall model results (i.e. the grand effect of "selection regime" and the interactions between "selection regime" and "time", Table 3) do not support our expectation of an evolved resistance against the fungal competitor in the FS populations. Also, after removing the non-significant "selection regime × time" interaction term no grand effect of selection emerged (*F*_1,4 _= 1.04, *p *= 0.3656). Given that there are only three populations for each selection treatment and the apparently lesser influence on fungal development of FS larvae compared to UC larvae (Figure 3), we performed a *post hoc *power analysis on the grand effect of the selection regime using GPower 3.1 [30]. Power analysis measures the probability that the statistical test will reject the null hypothesis, that the selection regime has no effect on fungal development, when it is false and the alternative hypothesis is correct. A test with a power greater than 0.8 (or committing a *type 2 error, β*≤0.2) is conventionally considered statistically powerful [31]. The power obtained, 1-*β *= 0.43 (on the *F*-test for "selection regime", with an effect size of 0.23) indicates that the power of this test is low. We should thus be skeptical with regard to not rejecting the null hypothesis.

**Table 3:** Suppression of fungal growth by *Drosophila *larvae

Effect	*F*	*p*
selection regime (FS vs. UC)	*F*_1,4 _= 1.57	0.2787
time (6 hours interval)	*F*_11,44 _= 43.46	< 0.0001
selection regime × time	*F*_11,44 _= 1.58	0.1384

Mixed model analysis for repeated measurement of *Aspergillus nidulans *colony cover as affected by *Drosophila melanogaster *larvae from the two different selection regimes and time.

FS: fungal selected *Drosophila *populations, UC: unselected control populations.

## Discussion

The vast majority of studies investigating genetic variation and adaptive evolution in antagonistic species interactions focused on host-parasite interrelationships, where natural enemies exploit host organisms for growth, reproduction and dispersal and where hosts evolve counter-adaptations (e.g. the internal immune system) to fend-off parasites or mitigate their negative effects on host fitness, e.g. [32,33]. Thus, the evolutionary response in our experiment is comparable to other studies that looked for heritable variation in protection against natural enemies, mainly macro-parasites and pathogens, in *D. melanogaster *[34-37]. However, it is comparatively new to consider non-parasitic microbes as potentially important competing antagonists of insects and other arthropods [38,39]. This consideration adds a novel level of selective pressure affecting life-history evolution.

Our first major finding is that selection pressure by *A. nidulans *resulted in *Drosophila *populations showing a higher probability of larvae surviving competition with this fungus. This result had been predicted by an earlier study and adds further evidence that there is heritable variation in the mechanisms underlying protection against competing filamentous fungi [25]. Thus, the genetic and hence the phenotypic structure of *Drosophila *populations may be subject to selection pressure through non-parasitic filamentous fungi. It is interesting that survival was not different in FS and UC *Drosophila *larvae when they were forced to develop in the presence of *A. flavus *and *A. fumigatus*. Thus, cross-protection against other fungi is not a consequence of selection pressure from *A. nidulans*, nor does protection evolved against *A. nidulans *lead to reduced ability to survive competition with *A. fumigatus *or *A. flavus*. This may indicate that the evolutionary costs of the improved performance of the FS insects are not traded off against reduced protection from other fungi the insects may interact with. Susceptibility to climatic stress (e.g. temperature, desiccation) and food shortage seem, rather, to be genetically correlated with protection evolved against fungal competitors [40]. These costs may provide a means of maintaining the genetic variation in protection against competing fungi that is required for the evolution of adaptation to fungal competitors.

We found a higher survival probability in FS larvae than in UC larvae when larvae were confronted with Sterigmatocystin, the most toxic compound formed by *A. nidulans*. Sterigmatocystin is a highly toxic polyketide metabolite that attaches to DNA strands, possibly the major reason for the strong cytotoxic and mutagenic effects of this compound [29]. The genes involved in Sterigmatocystin biosynthesis are organized in a gene cluster which is controlled by several regulatory switches. These switches may link the formation of secondary metabolites with other fungal life-history traits such as vegetative growth and reproduction [6]. The tight regulation of secondary metabolite gene expression may allow the fungus to adjust its chemical arsenal to variation in ecological conditions, e.g. competition with saprophagous insects. Thus, increased protection from *A. nidulans *in the FS lines correlates positively with reduced sensitivity to one of the most likely chemical weapons of the fungus. It is interesting that FS larvae were not better protected against Aflatoxin B1 or Ochratoxin than were UC larvae. This lack of cross-protection to other mycotoxins suggests that the positive correlation between protection against *A. nidulans *and its secondary metabolite Sterigmatocystin is a specific adaptation to the toxic secretion of this mold fungus. Protection from the toxic effects of Sterigmatocystin may arise through detoxification (e.g. by cytochrome P450 monooxygenases, [7]) of this compound or reduction of the damage it causes to the animals (e.g. more efficient DNA repair, [41]).

Evolved external defense against competing microbes, like defenses against parasites, can be divided into two conceptually different strategies [28]. First, selection may favor resistance, namely the ability to reduce competitor burden. This has a negative effect on the competing microbe because resistance aims at reducing or eliminating the fungal antagonist (competitive ability). As a consequence, FS larvae may impose direct costs on the fungus which in turn select for fungal counter-adaptations. Second, animals may evolve tolerance, the ability to reduce the damage inflicted by a given fungal competitor. Evolution of tolerance implies no changes in the negative consequences for the competing fungus and should therefore not result in antagonistic co-evolution [28].

Resistance or competitive ability may be associated with a behavioral defense. However, positive density-dependence in larval survival in the presence of competing mold [19] does not seem to be a mere by-product of gregarious egg-laying behavior by flies [42,43] and hence higher larval densities. In contrast, mold suppression is achieved by clumping of larvae in the active growth zone of fungi where they attack young exploitative hyphae [44]. Thus, in addition to the proposed physiological adaptation to the fungal toxins (see above), protection against mold may be mediated by the intensity of attack against *A. nidulans *increasing the insects' competitive ability. Increased survival of FS larvae on Sterigmatocystin contaminated substrate may point to the evolution of a compensatory mechanism rendering the fungal chemical defense less harmful to the insects. However, enhanced competitive ability may provide an additional benefit to the larvae or may even be the consequence of better protection against mycotoxins. However, larvae from the FS populations did not evolve increased resistance as fungal growth was not affected differently by larvae from the two selection regimes (Figure 3). The low power of the test statistics means, however, we cannot exclude that FS larvae had an even weaker negative effect on *A. nidulans*. This interesting correlated response to selection deserves further experiments. Thus, we suggest that improved larval development in the FS strains is due to increased tolerance of the fungal competitor possibly mediated by mechanisms reducing the toxic effect of fungal secondary metabolites.

The trend towards a less pronounced suppressing effect of FS than of UC larvae on *Aspergillus *colonies (Figure 3) might support the idea of a negative genetic correlation between resistance and tolerance [45,46] in *Drosophila *external defense against noxious microbes. Whether the evolution of tolerance of fungal competitors in saprophagous*Drosophila *larvae really exerts no selection pressure on the fungi remains to be investigated. Such investigations would require more detailed recording of fungal fitness consequences such as the effects of insects on fungal reproduction.

## Conclusion

This study on insect-fungus competition adds to our understanding of animal responses to antagonistic but non-pathogenic microbes, and hence provides further, albeit indirect, evidence of a critical role of toxic fungal secondary metabolites as a chemical shield against natural enemies [3]. Further experiments testing the effect of insect competition on the evolution of fungal life-history traits and secondary metabolites biosynthesis may help resolve this problem. Nonetheless, our experimental results support the notion that noxious filamentous fungi inhabiting *Drosophila *breeding sites have the potential to impose selection pressure that may explain the heritable variation in protection against competing fungi in field populations. Moreover, our study presents the first experimental evidence that the evolution of insect defense strategies against a toxic fungus involves developing mechanisms rendering less effective the chemical weaponry of fungi. However, instead of selection increasing resistance against fungi, it appears to have increased larval toleration of the fungal competitor. Therefore, adaptive changes in the insect populations in response to noxious mold do not increase selection pressure on the fungus to overcome this type of defense and hence should not fuel antagonistic co-evolution.

The evolutionary response we observed in our experimental study appears to be due to a specific adaptation of the insects to the fungus *A. nidulans *alone rather than selection for a more general response to fungal competitors. Thus, although a substrate generalist *D. melanogaster *may well be a specialist for interactions with competing fungi and their secondary metabolites. If this holds true for similar insect-microbe interactions and is successful in the face of the high diversity of noxious mold species the insects encounter under field conditions remains to be the subject to future studies.

## Methods

### Flies and fungi

We derived the *Drosophila melanogaster *starting population for the selection experiment from 113 isofemale lines collected from decaying plums in Kiel, Germany (ca. 54°N, 10°E) in August 2006. The population was kept at 25°C in large population cages with > 1000 individuals in each non-overlapping adult generation. Larvae developed at moderate densities on standard *Drosophila *cornmeal medium [19]. The population was allowed to adapt to the standard laboratory conditions for approximately one year (29 generations) before we started the selection experiment.

We used the common saprotrophic mold *A. nidulans *as the fungal competitor [40]. The fungus was cultured on malt extract agar at 25°C and a 14 hours photoperiod for approximately 4-5 days. Mature conidia (asexually produced spores) were washed off with saline solution (0.9% NaCl) containing the surfactant Tween 80 (0.1%) and stored at 4°C. Before inoculating the experimental units (see below) with conidia we adjusted the inoculate to a titer of 1000 conidia per microliter by using a haemocytometer (Neubauer^® ^improved).

### Selection protocol

The starting population was split into six lines. Three of these were subjected to selection (FS) and three were left unselected as control lines (UC). Development of *Drosophila *larvae from both the FS and the UC lines took place in autoclaved 2 ml micro tubes containing 1 ml sterile standard *Drosophila *medium. In order to allow fungal growth the medium was not treated with any antimicrobial agents. Ten freshly hatched, sterile larvae were transferred to each tube which was then sealed with a 10 mm autoclaved cotton plug (dental rolls, Celluron^®^, Paul Hartmann AG, Germany). We obtained sterile larvae from eggs that had been dechorionated using sodium hypochlorite (6%). This procedure ensured sterility by killing all the microorganisms that might influence insect-fungus competition. The dechorionated eggs were carefully washed in sterile water, transferred to a Petri dish containing a sterile agar layer and incubated at 25°C overnight. The larvae hatched by the following day and were transferred with a fine sterile brush into the experimental tubes. We avoided contamination by other microbes because other microbes, beneficial or otherwise, may influence the outcome of such an evolution experiment (e.g. mitigate the impact of *A. nidulans*).

Except for the first three insect generations where selected populations where confronted with *A. nidulans *every generation, see [40], we applied alternating cycles of selection and non-selection to the FS populations. The UC lines were always kept under mold-free conditions. For each selection cycle we set up 110-120 tubes for each line. During the first 17 generations, larvae from the FS lines were confronted with one day-old *A. nidulans *colonies (conidia-inoculated tubes were incubated at 25°C for ~24 hours before adding larvae), which caused moderate mortality (~34%) among the *Drosophila *larvae [40]. With this number of replicated tubes per population, we were able to keep the population size of both selection and control lines well above 200 individuals. This population size is enough to reduce the risks of inbreeding and genetic drift [47]. After generation 17 we intensified the selection pressure by giving *A. nidulans *a 'head-start' of two days (two selection cycles) or three days (three selection cycles). The two day advantage caused ~65% mortality in larvae from the base population and the three day advantage caused 80% mortality. Given this significantly higher larval mortality, we adjusted the number of experimental tubes accordingly to avoid an unwanted drop in the population size in the FS lines. After each selection and non-selection step, the new generation of adult *Drosophila *from both the selected and unselected lines were kept in small population cages (0.005 m^3^). Flies were fed *ad libitum *with a yeast-hydrolysate and sucrose mixture and provided with water for five to seven days before they were allowed to lay eggs to establish the next larval generation. For the non-selection steps we cultured the larvae on standard *Drosophila *medium in 0.000165 m^3 ^vials at moderate larval densities.

After generation 26 (14^th ^selection cycle), we stopped fungal selection pressure and kept all fly populations under non-selection conditions. Two generations later we started testing for direct responses, i.e. evolved protection against *A. nidulans*, and correlated responses, i.e. protection from other fungi, sensitivity to mycotoxins and effects on fungal development.

### Direct response - protection against *A. nidulans*

To test for evolved protection against the fungal competitor we quantified survival on *A. nidulans *infested substrate of *Drosophila *larvae from both the FS and UC lines. We prepared the larvae and experimental tubes using the same protocol as for a selection cycle (see above). Ten larvaefrom each population were transferred to a single experimental tube harboring two-day old fungal colonies. Twenty replicated tubes were established for each population. In parallel, we checked for line specific variation in larval survival under mold-free conditions (20 replicates of each population).

For all setups, the emergence of adult flies was recorded daily. We considered a single experiment as finished when no adult flies emerged for three days or more. Tests for evolved protection against *A. nidulans *were performed three times after we stopped selecting.

### Correlated responses I - cross-responses to other fungi

Cross-responsiveness was tested by confronting 10 larvae per tube from the FS or UC lines with *A. fumigatus *or *A. flavus *(*n *= 20 for each population and each fungal species). Conidia from *A. fumigatus *were pre-incubated for ~24 h before larval transfer. In the *A. flavus *treatment, larvae were added immediately after inoculation of the substrate with conidia. One thousand conidia were inoculated for both fungal species. Adult fly emergence was recorded as above.

### Correlated responses II - sensitivity to Sterigmatocystin and other mycotoxins

The fungal secondary metabolites Sterigmatocystin, Aflatoxin B1 and Ochratoxin A were obtained from Sigma Aldrich, dissolved in acetone and stored at -20°C. Each experimental tube contained the same amount of larval food medium as in all the other experiments. Ten microlitres of the mycotoxin solution was pipetted onto the medium to give final concentration of 1, 2 or 3 μg mycotoxin per milliliter substrate, depending on the experiment (Figure 2 in Result section).

Ten microliters pure acetone was pipetted into the control tubes (no mycotoxins). The acetone was allowed to evaporate for three hours before 10 first instar larvae were transferred to each tube. We set up 10 replicates for each combination of population, toxin and concentration. Confrontation with Sterigmatocystin was performed for the first time at generation 30 and a second time together with Aflatoxin B1 and Ochratoxin A in generation 33. Fly emergence was recorded as before.

### Correlated responses III - effects on fungal performance

To test whether larvae from the FS and the UC lines differently affect fungal growth, we confronted two-day old *A. nidulans *colonies with ten *Drosophila *larvae using our standard experimental tubes (n = 10 replicates for each treatment-population combination). Twenty-four hours after larval transfer we started taking digital images (Canon^® ^X100S, 8MPX) of the substrate surface every six hours. This allowed us to measure the substrate area covered by fungal tissue relative to the total surface (with ImageJ: http://rsbweb.nih.gov/ij/). In order to quantify the effect of insect competition on fungal growth we randomly assigned tubes containing both fungi and larvae to independent tubes where fungi grew without insect competitors. Relative to the size of undisturbed fungal colonies, for each pair of experimental tubes, we subtracted the proportionof the substrate area covered by undisturbed colonies from the area covered by fungi that had been challenged by larvae.

Assuming that the insect larvae hamper fungal growth [19], we expected to obtain Δ fungal cover values smaller than zero. The stronger the effect of larvae the closer the Δ fungal cover value would approach -1, whereas a value of zero would indicate no effect of insect competition on fungal colony growth.

### Statistical analysis

We used the GLMMIX procedure of SAS 9.0 with a logit link function and binomial error distribution to fit a generalized linear model that allows including random factors [40]. 'Selection regime' (FS, UC) was specified as the fixed factors.'Fly lines' were nested within 'selection regime' and interactions with other factors were considered random factors. Mold growth patterns were analyzed as repeated measures with MIXED procedure of SAS 9.0. Each experimental unit was considered as a repeated subject. Since the different levels of the repeated effect represented different time steps, we fitted a time series model by using the autoregressive 1 covariance structure, AR(1) [48].

## Authors' contributions

MR conceived the study and designed the experiments. MT carried out the experiments. MR and MT analyzed the data and wrote the manuscript. Both authors read and approved the final version of the manuscript.
